# Variations in implementation of antimicrobial stewardship via telehealth at select Veterans Affairs medical centers

**DOI:** 10.1017/ash.2023.268

**Published:** 2023-09-29

**Authors:** Geneva Wilson, Amanda Vivo, Daniel Livorsi, Rabeeya Sabzwari, Christopher Crnich, Robin Jump, Charlesnika Evans

## Abstract

**Background:** Antimicrobial stewardship programs (ASPs) seek to reduce the prevalence of antimicrobial-resistant and healthcare-associated infections. There are limited infectious disease (ID) physicians and pharmacists to support these ASPs, particularly in rural areas. The Veterans Health Administration has a robust telehealth program in place. Our previous work has demonstrated the feasibility of using telehealth modalities to support ASPs at rural Veterans Affairs medical centers (VAMCs) by pairing them with an ID expert from a larger, geographically distant, VAMC. This program, dubbed the Videoconference Antimicrobial Stewardship Team (VAST), emphasizes discussion of patients undergoing treatment for an active infection and additional relevant clinical topics with a multidisciplinary team at the rural VA. VAST implementation is ongoing at VAMCs. To understand and compare the qualitative differences in implementation, we used process maps to describe the VAST at 3 VAMC dyads. **Methods:** Team members from each dyad participated in interviews at 3, 6, and 9 months after beginning their VAST sessions. Questions addressed several aspects of VAST implementation and included identifying cases and topics to discuss; advance preparation for meetings; the frequency and general structure of VAST meetings; and documentation including workload capture. The research team used the responses to develop process maps to permit visual display and comparison of VAST implementation. **Results:** The first dyad began in January 2022 and the third in March 2022. The sessions had 3 phases: preparation, team meeting, and documentation of experts’ recommendations. Tasks were shared between VAST champions at the rural VAMC and the ID experts (Fig. 1). The preparation phase showed the most variation among the 3 dyads. In general, champions at the rural VA identified cases and topics for discussion that were sent to the ID expert for review. The approaches used to find cases and the type of preparatory work by the ID expert differed. Team meetings differed in both frequency and participation by professionals from the rural site. Documentation of expert recommendations processes appeared similar among the dyads. **Discussion:** Each of the 3 dyads implemented VAST differently. These results suggest that the overall structure of the VAST is readily adaptable and that each site tailored VAST to suit the clinical needs, workflow, and culture of their partner facility. Future work will seek to determine which aspects in the preparation, team meeting, or documentation phases are associated with successful ASPs, including assessment of quantitative and qualitative outcomes.

**Disclosures:** None

